# Editorial: Gaze and postural stability rehabilitation

**DOI:** 10.3389/fneur.2022.1034012

**Published:** 2022-10-21

**Authors:** Leonardo Manzari, Nicolas Perez-Fernandez, Marco Tramontano

**Affiliations:** ^1^MSA ENT Academy Center, Cassino, Italy; ^2^University Clinic of Navarra Pamplona, Navarra, Spain; ^3^Fondazione Santa Lucia IRCCS, Rome, Italy; ^4^Department of Movement, Human and Health Sciences, University of Rome “Foro Italico”, Rome, Italy

**Keywords:** vestibular, visual, proprioceptive, gaze, postural, assessment, rehabilitation

Proprioceptive, Visual, Vestibular, and Cognitive systems interact in a continuous sensorial re-weighting, ensuring gaze and postural control (1, 2). The central nervous system integrates the information originating from these systems into a continuous sensorial re-weighting that ensures postural control in both static and dynamic conditions (3, 4). The contribution of each sensory system changes depending on environmental conditions and the motor task performed by the person (5–7). To tailor a rehabilitative program for patients with gaze and postural stability disorders, a multidimensional assessment is required. A wide range of both clinical and instrumental evaluations could be performed before the rehabilitative approach in order to obtain quantitative and qualitative information about the patient's balance and gait disorders, supporting the rehabilitative staff in designing the most suitable therapeutic intervention. Instrumental assessment of the vestibular system has made significant progress in recent years. Two protocol tests are available in the clinical practice to evaluate the Vestibular Ocular Reflex (VOR) function through the use of Video Head Impulse Test (vHIT): Head Impulse Paradigm (HIMP) and Suppression Head Impulse Paradigm (SHIMP) (8–10). The head turn stimulus and the eye movement recording are identical. All that is changed are the instructions—from “look at that fixed target on the wall” to “look at the moving target.” At the same time, vestibular-evoked myogenic potentials are the most suitable test to evaluate otolith functions in patients with unilateral vestibular hypofunction in the acute and sub-acute phases (11, 12).

An innovative evaluation strategy could be represented by the inertial measurement unit sensors (IMU)-based assessment that provides valid objective metrics able to discriminate, with a higher sensitivity than clinical scores, between healthy people and patients with multiple sclerosis (MS) (Carpinella et al.) and in other neurological conditions (13). This approach would help in tracking these impairments over time and identifying those individuals who may benefit from preventive motor exercise and better tailor the rehabilitative program.

Standard rehabilitation, aimed at the recovery of static and dynamic postural stability, is usually focused on trunk stabilization and on exercises consisting of maintaining the standing position on an unstable platform such as oscillating boards and foam cushions (14). Another useful strategy is to work on postural control excluding the visual feedback and stimulating the sensory reweighting. Moreover, it could be effective to train the dynamic gait stability using a mechanism commonly required in daily life, defined as the dual-task paradigm (5). These exercises consist of combining a walking task with a cognitive one.

The gaze stability exercises consist of holding the gaze on a firm target during active horizontal and vertical head movements (15) or stimulating the refixation saccades (16). Another interesting strategy could be Galvanic vestibular stimulation (GVS) which can increase or decrease the firing rate of vestibular afferents by reversing the polarity. The cathodal galvanic stimulation results in excitation and the anodal galvanic stimulation results in the inhibition of the vestibular afferents through the spike trigger zone of primary afferents (Tohyama et al.). The stimulation of the vestibular system using bipolar GVS has an influence on visual vertical perception and standing posture depending on the polarity of the stimulation and hemispheric lesion side (Tohyama et al.). Furthermore, the noisy GVS (nGVS) can modulate the VOR-gain (Matsugi et al.). This will improve the understanding of the neural mechanisms that underlie balance disorders and the development of effective therapy and rehabilitation in the future.

An interesting contribution was the study of the trunk muscle activation patterns during the turning of patients with stroke. Indeed, the results of this trial (Chen et al.) provide insights into the contribution and importance of the trunk muscles during turning and the association with turning difficulty after stroke, which can guide the development of more effective rehabilitation therapies. Technological devices could be used in support of conventional therapy in the recovery of gaze and postural stability disorders. Different devices were used with a positive effect on postural stability in neurological disorders. Among these, are virtual reality, augmented reality (Cerritelli et al.), and load auditory feedback in people with neurological disorders (Tamburella et al.).

The articles in this Research Topic are focused on but not limited to the evaluation of the gaze and postural function in both static and dynamic conditions, and on the new rehabilitation strategies for balance disorders. These studies provide a snapshot of issues relevant within the neuro-otologic field. They provide new small, but essential, steps in advancing knowledge to better design further studies for the evaluation and treatment of balance disorders.

As editors of all these articles, we would like to encourage the readers to take their time to read these articles and to update their knowledge on these topics.

## Author contributions

Writing—original draft preparation: MT. Writing—review and editing: LM and NP-F. All authors listed have made a substantial, direct, and intellectual contribution to the work and approved it for publication.

## Conflict of interest

The authors declare that the research was conducted in the absence of any commercial or financial relationships that could be construed as a potential conflict of interest.

## Publisher's note

All claims expressed in this article are solely those of the authors and do not necessarily represent those of their affiliated organizations, or those of the publisher, the editors and the reviewers. Any product that may be evaluated in this article, or claim that may be made by its manufacturer, is not guaranteed or endorsed by the publisher.

## References

[1] Bruijn SM van Dieën JH. Control of human gait stability through foot placement. J R Soc Interface. (2018) 15:20170816. 10.1098/rsif.2017.0816 29875279 PMC6030625

[2] Tramontano M Piermaria J Morone G Reali A Vergara M Tamburella F. Postural changes during exteroceptive thin plantar stimulation: the effect of prolonged use and different plantar localizations. Front Syst Neurosci. (2019) 13:49. 10.3389/fnsys.2019.00049 31572134 PMC6753192

[3] Feller KJ Peterka RJ Horak FB. Sensory re-weighting for postural control in Parkinson's disease. Front Hum Neurosci. (2019) 126:31057379. 10.3389/fnhum.2019.00126 31057379 PMC6478764

[4] Tramontano M. Dell'Uomo D Cinnera AM Luciani C Di Lorenzo C Marcotulli M Vona F Mercuro A Abbruzzese S. Visual-spatial training in patients with sub-acute stroke without neglect: a randomized, single-blind controlled trial. Funct Neurol. (2019) 34:7–13. 31172934

[5] Tramontano M Morone G Curcio A Temperoni G Medici A Morelli D. Maintaining gait stability during dual walking task: effects of age and neurological disorders. Eur J Phys Rehabil Med. (2017) 53:7–13. 10.23736/S1973-9087.16.04203-9 27575014

[6] Tramontano M Bonnì S Martino Cinnera A Marchetti F Caltagirone C Koch G. Blindfolded Balance Training in Patients with Parkinson's Disease: A Sensory-Motor Strategy to Improve the Gait. Parkinsons Dis. (2016) 2016:7536862. 10.1155/2016/7536862 26977334 PMC4763005

[7] Bent LR McFadyen BJ Inglis JT. Visual-vestibular interactions in postural control during the execution of a dynamic task. Experim Brain Res. (2002) 146:490–500. 10.1007/s00221-002-1204-8 12355278

[8] Manzari L Tramontano M. Suppression Head Impulse Paradigm (SHIMP) in evaluating the vestibulo-saccadic interaction in patients with vestibular neuritis. Eur Arch Otorhinolaryngol. (2020) 277:3205–12. 10.1007/s00405-020-06085-6 32472160

[9] Manzari L Princi AA De Angelis S Tramontano M. Clinical value of the video head impulse test in patients with vestibular neuritis: a systematic review. Eur Arch Otorhinolaryngol. (2021) 278:4155–67. 10.1007/s00405-021-06803-8 33893851

[10] Manzari L De Angelis S Princi AA Galeoto G Tramontano M. The clinical use of the suppression head impulse paradigm in patients with vestibulopathy: a systematic review. Healthcare. (2022) 10:1182. 10.3390/healthcare10071182 35885709 PMC9320756

[11] González JCF Barreiro SB. Pérez Fernández N Vanspauwen R Ramos-Macias A. Differences in vestibular-evoked myogenic potential responses by using cochlear implant and otolith organ direct stimulation. Front Neurol. (2021) 12:663803. 10.3389/fneur.2021.663803 34113311 PMC8185293

[12] Manzari L Graziano D Zambonini G Faralli M Morone G Tramontano M. The clinical course of vestibular neuritis from the point of view of the ocular vestibular evoked myogenic potential. J Laryngol Otol. (2022) 136:129–36. 10.1017/S0022215122000081 35001866

[13] Belluscio V Bergamini E Tramontano M Orejel Bustos A Allevi G Formisano R. Gait quality assessment in survivors from severe traumatic brain injury: an instrumented approach based on inertial sensors. Sensors. (2019) 19:5315. 10.3390/s19235315 31816843 PMC6928771

[14] Bonnì S Ponzo V Tramontano M Martino Cinnera A Caltagirone C Koch G. Neurophysiological and clinical effects of blindfolded balance training (BBT) in Parkinson's disease patients: a preliminary study. Eur J Phys Rehabil Med. (2019) 55:176–82. 10.23736/S1973-9087.18.05126-2 29745627

[15] Tramontano M Russo V Spitoni GF Ciancarelli I Paolucci S Manzari L. Efficacy of vestibular rehabilitation in patients with neurologic disorders: a systematic review. Arch Phys Med Rehabil. (2021) 102:1379–89. 10.1016/j.apmr.2020.11.017 33383031

[16] Matiñó-Soler E Rey-Martinez J Trinidad-Ruiz G Batuecas-Caletrio A Pérez Fernández N A. new method to improve the imbalance in chronic unilateral vestibular loss: the organization of refixation saccades. Acta Otolaryngol. (2016) 136:894–900. 10.3109/00016489.2016.1172730 27109262

